# Well-Differentiated Squamous Cell Carcinoma: Is Histological Differentiation a Relevant Prognostic Parameter?

**DOI:** 10.5826/dpc.1102a34

**Published:** 2021-04-12

**Authors:** Iago Gonçalves Ferreira, Ana Letícia Boff, Laura Luzzato, Paulo R. Martins Souza, Mariele Bevilaqua

**Affiliations:** 1 Dermatology Service, Santa Casa de Misericórdia de Porto Alegre, Brazil; 2 Federal University of Health Sciences of Porto Alegre, Brazil

**Keywords:** squamous cell carcinoma, head and neck squamous cell carcinoma, skin neoplasms, prognosis

## Introduction

Squamous cell carcinoma (SCC) represents about 20% of non-melanoma skin cancers, being the second most prevalent type after basal cell carcinoma [1,2]. Most SCCs have good prognosis after surgical excision. However, about 5% of cases progress to locally advanced or metastatic lesions with unfavorable prognosis [3,4]. The degree of histological differentiation has been considered an independent predictor of recurrence, metastasis, and survival rates. Lower recurrence and metastasis rates are attributed to well-differentiated SCCs as compared to poorly differentiated SCCs [1]. We present the case of a patient with well-differentiated SCC of the head, with an exuberant clinical presentation.

## Discussion

**Figure 1:**
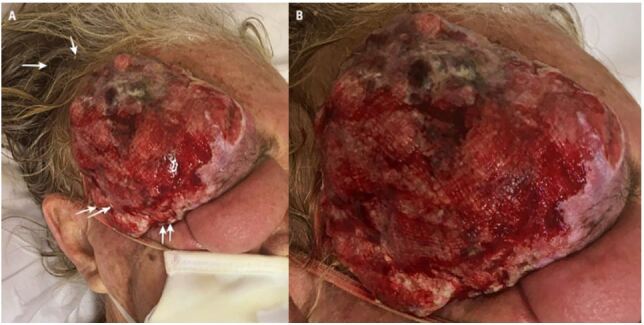
(A, B) Macroscopic clinical images showing the tumor on the patient’s face and the presence of myiasis larvae (white arrows).

A 78-year-old female patient from a rural town in Southern Brazil was referred to the Dermatology Service for a tumoral lesion in the right upper face. The patient was a farmer and smoker, had diabetes and a history of breast cancer, and had skin phototype III. The tumor had progressively grown for about 2 years, with intensive expansion in the past 6 months. The patient reported previous secondary bacterial infections, which were treated with systemic antibiotics, and myiasis in the lesion. She had previously refused tumor biopsy. The physical exam showed an extensive, friable tumor with necrotic areas, bloody drainage and myiasis larvae on the lesion surface (Figure 1).

**Figure 2:**
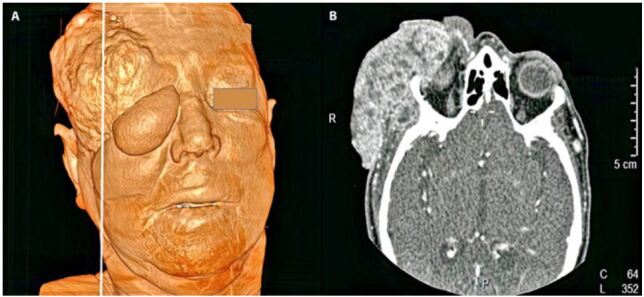
(A) A 3D tomographic image reconstruction showing an expansive lesion in the right frontal area with periorbital involvement. (B) Contrasted computed tomographic image showing an expansive lesion in the right frontal area, in the axial slideplane.

Due to the advanced condition, the patient was referred to the Emergency Service for hospital admission. Cranial computed tomography showed a tumor mass in right upper face with invasion of the subcutaneous tissue and skullcap (Figure 2).

**Figure 3:**
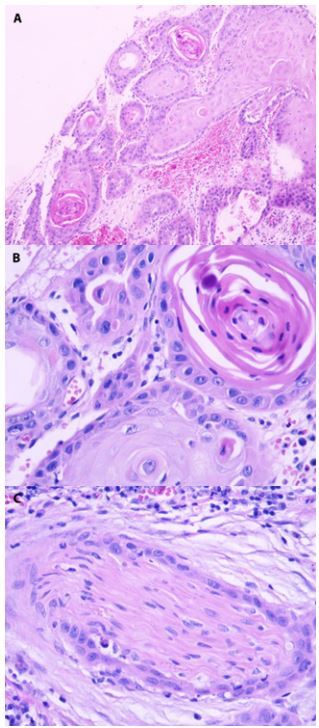
(A) Squamous cell proliferation infiltrating the dermis (H&E, ×40). (B) Keratin production in the center of the squamous proliferation (H&E, ×400). (C) Perineural infiltration by neoplastic cells (H&E, ×400).

The patient underwent surgical resection of the tumor and of the frontal process of zygomatic bone, followed by skin grafting from an abdominal donor site. The surgical sample was submitted to histological analysis, which showed a well-differentiated SCC with an infiltrative, ulcerated pattern, invasion of the dermis, hypodermis, periocular soft tissues, bone, and fibrous tissue, as well as perineural invasion (Figure 3). Clinical staging did not demonstrate metastasis, and the patient evolved with bleeding at the surgical site and secondary anemia. After clinical stabilization, she was discharged from the hospital with outpatient follow-up. Due to her comorbidities (chronic kidney failure, smoking and type 2 diabetes) as well as her difficulty adhering to treatment, the oncology team opted for palliative management of the case.

The prognosis of SCCs depends on factors intrinsic to each patient and cancer, which will determine the aggressive potential of a lesion. The risk factors for poor prognosis are related to size and depth, extension to subcutaneous tissue, poor histological differentiation and perineural invasion. Regarding the individual, characteristics such as immunosuppression and previous SCCs are also considered [5,6].

Regarding histology, SCCs are classified according to their degree of keratinization, nuclear atypia and histological architecture. They are therefore classified into 4 degrees of differentiation: well differentiated (G1), moderately differentiated (G2), poorly differentiated (G3), and undifferentiated (G4) [1,7,8]. However, according to the National Comprehensive Cancer Network (NCCN), there is currently a tendency to classify SCCs into 2 degrees of differentiation: well to moderately differentiated, and poorly differentiated [9].

The NCCN’s Clinical Practice Guidelines in Oncology indicate that well-differentiated SCCs have better prognosis than poorly differentiated carcinomas, as well as lower rates of recurrence and metastasis (based on retrospective studies) [9]. Furthermore, the guidelines consider, as factors for poor SCC prognosis: neoplasms originating from chronic skin ulcers and scars; perineural involvement and poor histological differentiation; immunosuppressive status; adenoid, desmoplastic and adenosquamous histological subtypes; and size, with risk depending on tumor extension and the affected region [10].

Nevertheless, the prognostic parameters for SCCs are not always well-defined in the literature, which makes it difficult to elucidate which combinations of factors predict better or worse prognosis [5]. Some authors have pointed to poor histological differentiation as an important definer of metastasis and recurrence, and to tumor diameter and depth as parameters inversely proportional to differentiation grade in SCCs [1,7,11,12].

A French cohort study about advanced SCC (stage IV) showed that about 78% were well differentiated (G1) [13]. A cohort study with 195 German patients with stage III or IV SCCs demonstrated that about 80% did not have desmoplasia and 92% did not have neural invasion, diverging from factors for poor SCC prognosis [14].

We reinforce the understanding that the degree of differentiation should not be analyzed in isolation to express the impact of SCCs [6], as the patient had a lesion expansion of about 15 cm in diameter, with perineural invasion of dermis and hypodermis, facial disfigurement, and rapid, progressive growth, despite the well-differentiated degree of the tumor. Advanced head and neck SCCs may have an intense negative impact on the social interactions and functionality of individuals due to the facial disfigurement caused, and therefore are a risk factor for depression and suicide [6].

However, non-melanoma skin cancers have high cure rates, especially when diagnosed and treated early. Late diagnosis has been attributed to characteristics such as low social status, lack of personal hygiene, fear of diagnosis, and potential consequences [15]. Such situations were identified in this patient’s history: she was from a rural town and had bad memories from her previous breast cancer treatment. Thus, she refused to seek medical attention, which aggravated her condition.

This study draws attention to the fact that we must evaluate the prognostic histological parameters of cutaneous SCCs together with each patient’s unique factors. In isolation, histological differentiation should not be taken as a predictor of neoplastic behavior.
